# F6. PSYCHOLOGICAL RESILIENCE TO SUICIDAL THOUGHTS AND BEHAVIOURS: A QUALITATIVE EXAMINATION OF THE EXPERIENCES OF PEOPLE WITH MENTAL HEALTH PROBLEMS ON THE SCHIZOPHRENIA SPECTRUM

**DOI:** 10.1093/schbul/sby017.537

**Published:** 2018-04-01

**Authors:** Kamelia Harris, Patricia Gooding, Gillian Haddock, Sarah Peters

**Affiliations:** University of Manchester

## Abstract

**Background:**

Suicide is one of the leading causes of premature death in schizophrenia. The lifetime risk of death by suicide in people with schizophrenia is around 10 per 100 people. Some people can counter the impact of suicide risk (e.g., life stressors) by incorporating personal skills and experiences. Although exposure to life stressors can have a detrimental effect on mental health, this does not apply to everyone, as some individuals appear protected from the impact of stress. The notion of resilience in mental health has recently gained research attention. Resilience has been defined as an individual’s skills or resources that help them manage suicidal thoughts and feelings. Consistent with this definition, it is important to understand the psychological factors that contribute to resilience to the frequency and severity of suicidal thoughts and behaviours. We aimed to examine the experiences of psychological resilience to suicidal thoughts and behaviours of people with schizophrenia-spectrum mental health problems.

**Methods:**

We conducted face-to-face interviews with 20 individuals with experiences on the schizophrenia spectrum and lifetime experiences of suicidal thoughts, plans, and behaviours. Participants were recruited from community mental health services in the North-West of England, UK. A topic guide for semi-structured interviews was used to facilitate participants’ descriptions of their experiences in their own words. All interviews were audio-recorded, with participants’ consent, and transcribed verbatim. Thematic analysis was used to examine the themes and patterns within the data which were important in addressing the research aim.

**Results:**

The majority of the participants were white British, single, and living alone. The average age of the sample was 48 years (SD = 14.4; range: 23–75 years) and 50% were female. Several factors were involved in promoting psychological resilience. Participants described strategies that indicated they were being active in maintaining and developing their resilience. These included cognitive reasoning (e.g., rationalising and validating experiences) and active coping strategies (e.g., acceptance of experiences, perseverance). Perceived social support from significant others and mental health professionals (e.g., care coordinators, psychiatrists) also played an important role in bolstering individual resilience.

**Discussion:**

This is the first study to showcase the experiences of resilience to suicidal thoughts and behaviours from the unique perspective of individuals with mental health problems on the schizophrenia spectrum and a range of suicidal experiences (e.g., ideation, attempts, plans). The data indicated that resilience to suicidal thoughts and behaviours developed over time, through the experience of managing psychotic symptoms and their deleterious impact on individual wellbeing. People employed different types of coping strategies, depending on the severity of their psychotic symptoms and suicidal feelings. However, if their symptoms became severe, they usually sought support from mental health services. Efforts to develop psychological resilience to suicidal thoughts and behaviours in individuals with schizophrenia are of paramount importance to clinical research and practice. It is important to consider the impact of psychotic symptoms, which were the main precipitating factors of suicidal thoughts and behaviours for most participants, in the development of psychological resilience. Of note, only individuals under the care of mental health services were recruited into the study. Future research should explore the resilience experiences of people not accessing mental health services.

